# Determination of risk factors for fever after transarterial chemoembolization with drug-eluting beads for hepatocellular carcinoma

**DOI:** 10.1097/MD.0000000000027636

**Published:** 2021-11-05

**Authors:** Jinpeng Li, Congcong Shi, Jutian Shi, Jinlong Song, Nan Wang

**Affiliations:** a Intervention Ward One, Shandong Cancer Hospital and Institute, Shandong First Medical University and Shandong Academy of Medical Sciences, Jinan, Shandong, China; b Shandong Mental Health Center, Jinan, Shandong, China; c Department of Minimally Invasive Oncology of The First Affiliated Hospital of Shandong First Medical University, Jinan, Shandong, China.

**Keywords:** DEB-TACE, efficacy, fever, hepatocellular carcinoma, risk factors, TACE

## Abstract

This study was to identify risk factors affecting postembolization fever (PEF) of CalliSpheres drug-eluting bead transarterial chemoembolization (DEB-TACE) in the treatment of primary hepatocellular carcinoma (HCC).

One hundred eighty-eight consecutive patients with HCC who underwent DEB-TACE with fever between June 2017 and May 2019 were included in this retrospective study. The patients were divided into 4 groups based on the severity of posttransarterial chemoembolization (TACE) fever according to the degrees of body temperature. Univariate analysis and multivariate logistics regression were performed to identify potential risk factors for post-TACE fever.

In the stepwise multiple regression analysis, pre-TACE blood urea, small particle size and Cental liqefction (*P* < .05) were independent risk factors of severe post-TACE fever (*P* < .05, respectively). Portal vein thrombosis (*P* < .01), Child-Pugh stage (*P* < .01), and cycles of DEB-TACE (*P* < .05) were independent risk factors for clinical death, PEF was not associated with clinical death (*P* = .754) and 6-month survival (*P* = .524) in the univariate analysis. Moreover, multivariate Cox regression was performed, and Child-Pugh stage (B vs A) (*P* = .040) and portal vein thrombosis (yes vs no) (*P* = .033) were independent factors predicting unfavorable overall survival in HCC patients.

Pre-TACE blood urea, small particle size, and Cental liqefction were significantly correlated with the occurrence fever after DEB-TACE. Therefore, these factors should be taken into full consideration for the relief of fever. However, PEF after D-TACE was not associated with clinical death and 6-month survival rate.

## 1 Introduction

The incidence of hepatocellular carcinoma (HCC) has increased rapidly and become second killer of cancer-induced deaths. Generally, the majority of patients are diagnosed at middle or late stage and resulted in a low overall 5-year survival rate.^[1]^ Transarterial chemoembolization (TACE) has been recommended as first-line therapy for HCC and also provide survival benefit.^[2]^ The most frequent complication are relatively mild and tolerated by most patients, including fever, nausea, vomiting, and abdominal pain.^[3]^ The foreign literature reported that 86% of patients had fever after operation, while domestic literature statistics were 70.6%.^[4]^ Long-term high fever not only increases the patient's hospital stay and medical costs, but also affects the efficacy of TACE treatment of liver cancer and life quality.^[5]^ Therefore, studying the predictors of postinterventional fever in patients with HCC is of great practical importance to improve the quality of survival and reduce the number of hospital days and the cost of treatment.^[6]^ Therefore, the aim of our study was to assess the efficacy, safety, and prognostic factors of drug-eluting bead transarterial chemoembolization (DEB-TACE) in Chinese HCC patients.

The aim of this study was to investigate the risk factors associated with fever in HCC patients after DEB-TACE.

## 2 Materials and methods

### 2.1 Patients

A total of 188 consecutive patients in Shandong Cancer Hospital and Institute with unresectable hepatic malignancy who underwent DEB-TACE and had post-TACE fever were included in this retrospective study between June 2017 and May 2019. The inclusion criteria of DEB-TACE were as follows: Eastern Cooperative Oncology Group performance status <2; international normalized ratio <1.5 and platelet count >50,000/mm^3^; compensated liver function (Child-Pugh class A or B); no refractory ascites or renal failure; no fever or infection before operation. The exclusion criteria were as follows: Patients who had infection within 1 week before TACE operation; TACE combined with other operations simultaneously; periodic use of antibacterial drugs to prevent infection. The study was approved by the Ethics Committee of Shandong Cancer Hospital [Ethical approval number: SDZLEC-2017-001-01]. All the patients or their legal guardian provided the written informed consents.

Finally, a total of 188 patients with unresectable HCC were included (128 males and 60 females, with a mean age of 54.62 ± 11.25 years).

### 2.2 Treatment

After routine pre-operative preparation, the femoral artery was punctured using the Seldinger technique, and the superior mesenteric artery of the celiac artery was firstly imaged to assess the blood flow characteristics of the hepatic artery, the blood supply to the tumor, and the patency of the portal vein before selective cannulation to the tumor supplying artery and injection of CalliSpheres drug-loading microspheres (Jiangsu Hengrui Medicine Co. Ltd., Jiangsu, China) (300–500 μm or 100–300 μm). In case arteries supplying the tumors were not developed, arteriography was continued of the superior mesenteric artery, bilateral inferior phrenic arteries, internal thoracic arteries, and the aortic suprarenal artery to confirm these arteries supplying the tumors. Then, a 2.7F micro-catheter (Terumo, Japan) was advanced super-selectively to the supplying artery of the tumor. CalliSpheres beads were fully loaded Epirubicin at a dosage of 60 to 80 mg and mixed with Ioversol at a volume ratio of 1:1 to 1.2, followed by standing for 5 minutes. Before use, the sample was mixed and placed in a 1 mL injector. The bead diameter and injecting sequence depended on the tumor size and supplying vessels. Subsequently, the mixture was manually injected in a pulsed mode into the tumor-supplying artery at a rate of 1 mL/min under fluoroscopic monitoring until the developer was stable or approached stability in the arterial flow to the tumor which was the technical endpoint, whereas complete occlusion was avoided. Repeated hepatic arteriography was performed to assess devascularization after embolization. Chemoembolization was performed as superselectively as possible.

### 2.3 Data collection

In our study, fever after TACE is recorded in the nurse's body temperature record. PEF was defined as a body temperature greater than 38.0°C that developed within 3 days of TACE without evidence of infection. According to the degree of fever, patients with post-D-TACE fever were divided into 2 groups (patients with PEF vs patients without PEF). By reviewing the patient's medical records, the host-related variables, laboratory data, and radiological data of patients before D-TACE were collected to assess the potential risk factors for post-TACE fever. The host-related variables included age, sex, history of diabetes, Child-Pugh class, Eastern Cooperative Oncology Group status, liver cirrhosis or portal hypertension, dose of epirubicin administered, size of DEB used, and history of liver cancer resection or cholecystectomy. The laboratory data included serum alpha-fetoprotein, albumin, and total bilirubin levels, serum creatinine, and postoperative blood neutrophilic granulocytes and serum albumin. The radiological data included the presence or absence of PVTT, maximal tumor size, radiological findings (poorly defined or well defined), portal vein thrombosis, number of lesions, and CT response after 1^st^ TACE. They were blinded to the demographic and laboratory data, and not involved in the treatment. Poor blood glucose control was defined as a mean blood glucose level >200 mg/dL.

### 2.4 Efficacy evaluation

Pre- and postoperative CT or MRI images of patients were recorded to assess postoperative tumor response according to the modified response evaluation criteria in solid tumors (mRECIST) criteria; if the patient had HCC with multiple intra-hepatic metastases, the value of the diameter was the sum of all measurable tumor diameters. Complete remission (CR); partial remission (PR); progressive disease; stable disease. CR + PR was recorded as the objective remission rate (ORR). ORR = CR + PR.

### 2.5 Statistical analysis

SPSS 25.0 (SPSS Inc., Chicago) was used to analyze data. The data were compared between groups using the *t* test for 2 independent samples, and the data were compared between groups using the χ2 test. The Kaplan-Meier survival curves were plotted and log-rank test was used to analyze the long-term survival of the 2 groups, and Cox regression was used to analyze the factors influencing the long-term survival. *P* < .05 was considered statistically significant.

## 3 Results

### 3.1 Demographic and laboratorial characteristics

All DEB-TACE procedures achieved technical success according to the Society of Interventional Radiology guidelines. According to the degree of fever, there were 46 patients with no fever (<37.0°C) in group A, 78 patients with low fever (37.0–37.9°C) in group B, 48 patients with moderate fever (38.0–38.9°C) in group C, and 16 high fever (>39°C) in group D. The diagnosis of HCC was based on pathology (biopsy, n = 28) or on the American Association for the Study of Liver Practice Guidelines (n = 160). Of the 188 patients, 140 patients had a history of cirrhosis, and 48 patients were non-cirrhotic. The etiologies of liver cirrhosis were hepatitis B infection (n = 128, 68.1%) and hepatitis C infection (n = 12, 6.3%). There were 145 patients (77.1%) in Child-Pugh class A and 43 patients (22.9%) in Child-Pugh class B. The median serum total bilirubin was 18.6 μmol/L (IQR 11.5–24.4 μmol/L), and the median albumin was 35.3 g/L (IQR 30.4–36.0 g/L). Eighty patients (80/188, 42.6%) had PVTT, and 108 patients (108/188, 57.4%) did not have PVTT. Ninety-five patients (95/188, 50.5%) were treated with superselective TACE, 93 patients (93/188, 49.5%) were treated with non-superselective TACE (Table 1). The average length of stay among patients with fever was 6.1 ± 2.7, while without fever was 2.1 ± 0.9.

**Table 1:** The demographic and laboratorial characteristics of all patients.

Characteristics	
Age (yrs)	54.62 ± 11.25
Gender (male/female)	118/70 (62.7%/37.3%)
Child-Pugh class (A/B)	145/43 (77.1%/22.9%)
ECOG status (0/1)	157/31 (83.5%/16.5%)
Underlying liver disease (HBV/HCV/ none)	128/12/48 (68.1%/6.3%/25.6%)
Dose of epirubicin administrated (mg)	60 (IQR 60–80)
Size of DEB (100–300 μm/300–500 μm/500–700 μm)	48/135/5 (25.5%/71.8%/2.7%)
History of hepatectomy (presence/ absence)	15/173 (7.9%/92.1%)
Albumin (g/L)	35.3 (IQR 30.4–36.0)
Alpha-fetoprotein (>400 μg/L/≤400 μg/L)	87/101 (46.3%/53.7%)
Total serum bilirubin (μmol/L)	18.6 (IQR 11.5–24.4)
PVTT (presence/absence)	80/108 (42.6%/57.4%)
Superselective chemoembolization (presence/absence)	95/93 (50.5%/49.5%)

DEB = drug-eluting bead, ECOG = Eastern Cooperative Oncology Group.

### 3.2 Efficacy evaluation

**Figure 1:**
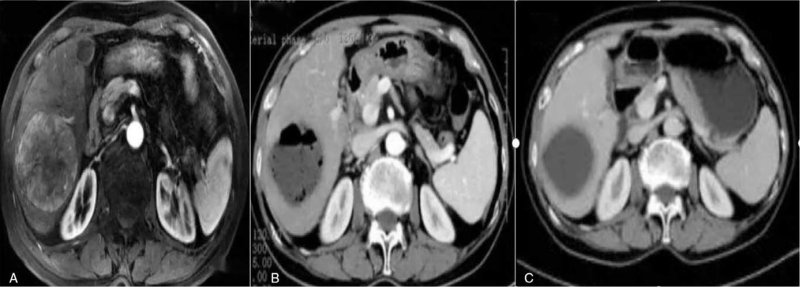
Male, 54 years old, HCC, BCLC A stage, refused surgical treatment and received D-TACE. (A) The specimens showed a 7.0 cm × 8.5 cm tumor at the right liver with significant enhancement in the arterial phase. (B) The patient developed chills and fever with a maximum temperature of 39.5°C 2 days after D-TACE, and was treated with anti-inflammatory, hepatoprotective and rehydration fluids for symptomatic management and resolved. Discharged after 6 days of hospitalization for observation. CT shows extensive liquefied necrosis within the tumor with a small amount of gas. (C) CT showed complete necrosis of the tumor in the right lobe of the liver at 1 month postoperative follow-up, mRECIST: CR. BCLC = Barcelona Clinic Liver Cancer, CR = complete remission, HCC = hepatocellular carcinoma, mRECIST = modified response evaluation criteria in solid tumors.

All CalliSpheres drug-loaded microsphere treatments were successfully conducted, and the technical success rate was 100%. All 188 patients were followed up, and efficacy was evaluated with the mRECIST criteria. Among all patients 26 patients with CR, 90 patients with PR, 30 patients with stable disease, 52 patients with progressive disease, ORR 61.70% (116/188), disease control rate 77.66% (146/188) (Table 2, Fig. 1)

**Table 2:** Follow-up results based on the mRECIST criteria.

Groups	n	CR	PR	SD
Group A	46	0	19	10
Group B	78	4	37	14
Group C	48	18	26	5
Group D	16	4	8	1

CR = complete remission, mRECIST = modified response evaluation criteria in solid tumors, PR = partial remission, SD = stable disease.

### 3.3 Association of PEF with clinical variables

A univariate analysis indicated that pre-TACE blood urea (*P* < .05), size of DEB (*P* < .05), PVTT (*P* < .01), and alanine aminotransferase value after TACE (*P* < .01) were possible risk factors correlated with postembolization fever (PEF) in patients with HCC. A multivariate analysis using logistic regression showed that pre-TACE blood urea (*P* < .05) and size of DEB (*P* < .05) were independent predictive factors of PEF (Table 2). PEF was not associated with clinical death (*P* = .754) and 6-month survival (*P* = .524) in the univariate analysis.

### 3.4 Clinical death

The univariate analysis revealed that tumor size (*P* < .01), portal vein thrombosis (*P* < .01), favorable CT response after TACE (*P* < .01), and number of lesions (*P* < .01) were possible risk factors correlated with clinical death (Table 3). A multivariate analysis using logistic regression showed that portal vein thrombosis (*P* < .01), Child-Pugh stage (*P* < .01), and cycles of DEB-TACE (*P* < .05) were independent risk factors for clinical death (Table 3).

**Table 3:** Potential risk factors for post-TACE fever.

					*P* value	
Characteristics	Group A (n = 46)	Group B (n = 78)	Group C (n = 48)	Group D (n = 16)	Univariate	Multivariate
Age (yrs)	55.38 ± 10.45	53.78 ± 11.23	54.98 ± 10.79	53.05 ± 11.27	.328	.19
Gender (male/female)	40/6 (86.9%/13.1%)	68/10 (87.2%/12.8%)	43/5 (89.6%/10.4%)	12/4 (75.0%/25.0%)	.519	–
Child-Pugh class (A/B)	38/8 (82.6%/17.4%)	59/19 (75.6%/24.4%)	38/10 (79.2%/20.8%)	10/6 (62.5%/37.5%)	.402	–
ECOG status 0/1	37/9 (80.4%/19.6%)	62/16 (79.5%/20.5%)	41/7 (85.4%/14.6%)	14/2 (87.5%/12.5%)	.774	–
Underlying liver disease HBV/HCV/none	30/2/14 (65.2%/4.3%/30.5%)	60/2/16 (76.9%/2.6%/20.5%)	28/6/14 (58.3%/12.5%/29.2%)	10/2/4 (62.5%/12.5/25.0%)	.175	–
PVTT (presence/absence)	10/36 (21.7%/78.3%)	36/42 (32.1%/67.9%)	23/25 (37.5%/62.5%)	11/5 (31.2%/68.8%)	.003	–
pre-TACE blood urea (mmol/L)	6.65 ± 5.68	5.23 ± 1.79	4.54 ± 1.38	4.60 ± 1.26	.025	
Largest tumor size (cm)	5.8 (IQR 3.8–9.7)	7.3 (IQR 4.8–11.4)	6.6 (IQR4.8–10.7)	6.5 (IQR 4.3–10.5)	*.031*	–
Tumor near liver capsule (presence/absence)	14/32 (30.4%/69.6%)	28/50 (35.9%/64.1%)	20/28 (41.7%/58.3%)	7/9 (43.7%/56.3%)	.648	–
AFP > 400 μg/L/≤400 μg/L	18/28 (39.1%/60.9%)	38/40 (48.7%/51.3%)	25/23 (52.1%/47.9%)	6/10 (37.5%/62.5%)	.517	–
Albumin (g/L)	35.6 (IQR 30.1–37.0)	33.1 (IQR 29.7–35.4)	32.3 (IQR29.6–34.8)	31.5 (IQR 28.8–33.7)	.412	–
Total bilirubin (μmol/L)	17.2 (IQR 11.0–23.2)	18.3 (IQR 12.5–27.4)	18.7 (IQR14.0–23.0)	19.1 (IQR 14.0–23.0)	.376	–
Tumor involved scope (left/right/bilobar)	3/37/6 (6.5%/80.4%/13.1%)	12/58/8 (15.4%/74.3%/10.3%)	8/36/4 (16.7%/75.0%/8.3%)	3/9/4 (18.7%/56.3%/25.0%)	.370	–
Number of lesions 1/2–3/>3	9/20/17 (19.5%/43.5%/37.0%)	26/30/22 (33.3%/38.5%/28.2%)	10/25/13 (20.8%/52.1%/27.1%)	4/8/4 (25.0%/50.0%/25.0%)	.503	–
Dose of epirubicin (mg)	60 (IQR 60–80)	60 (IQR 60–80)	70 (IQR 60–80)	80 (IQR 60–80)	.676	–
Size of DEB (μm) 100–300/300–500/500–700	8/35/3 (17.3%/76.1%/6.6%)	40/28/10 (51.2%/35.9%/12.9%)	22/18/8 (45.8%/37.5%/16.7%)	9/4/3 (56.2%/25.0%/18.8%)	.001	–
Superselective TACE (absence/presence)	18/28 (39.1%/60.9%)	40/38 (51.3%/48.7%)	25/23 (52.1%/47.9%)	10/6 (62.5%/37.5%)	.349	–
Increase of ALT value after TACE (presence/absence)	10/36 (21.7%/78.3%)	58/20 (74.4%/25.6%)	36/12 (75.0%/25.0%)	14/2 (87.5%/12.5%)	.001	–

AFP = alpha-fetoprotein, ALT = alanine aminotransferase, DEB = drug-eluting bead, ECOG = Eastern Cooperative Oncology Group, TACE = transarterial chemoembolization.

### 3.5 Overall survival

**Figure 2:**
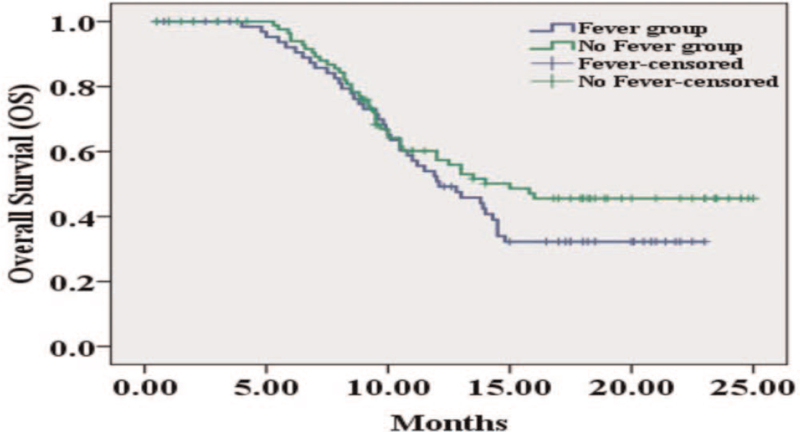
Comparison of OS between fever group and no fever group. OS = overall survival.

Comparison of overall survival (OS) between fever group and no fever group, the OS in the fever group was shorter than in the no fever group (12.1 m vs 15 m), but there was no significant statistical significance between the 2 groups (*P* = .192) (Fig. 2). As to factors affecting OS, univariate Cox regression disclosed that largest nodule size (≥5 cm vs <5 cm) (*P* = .045) and Child-Pugh stage (B vs A) (*P* = .030) predicted worse OS, while cycles of DEB-TACE (≥2 vs 1) (*P* = .036) predicted better OS in HCC patients. Moreover, multivariate Cox regression was performed, and Child-Pugh stage (B vs A) (*P* = .040) and portal vein thrombosis (yes vs no) (*P* = .033) were independent factors predicting unfavorable OS in HCC patients (Table 4).

**Table 4:** Multivariate analysis for postembolization fever and clinical death after D-TACE.

Variables	HR	95%CI	*P* value
Multivariate analysis^∗^			
pre-TACE blood urea	3.477	1.172–10.319	.025
Size of DEB	4.374	1.339–14.286	.015
Univariate analysis^†^			
Portal vein thrombosis	2.673	1.773–4.034	<.01
Number of lesions	3.242	1.029–10.215	.045
Child-Pugh stage (B vs A)	1.555	1.261–1.923	.006
Cycles of DEB-TACE (≥2 vs 1)	0.273	0.081–0.918	.036
Multivariate analysis^‡^			
Portal vein thrombosis (yes vs no)	2.490	1.078–5.751	.033
Child-Pugh stage (B vs A)	2.420	1.040–5.629	.040

DEB-TACE = drug-eluting bead transarterial chemoembolization, HR = hazard ratio, TACE = transarterial chemoembolization.

∗ Predictors for postembolization fever.

† Risk factors for clinical death.

‡ Predictors for clinical death.

## 4 Discussion

PEF is a common clinical manifestation of postembolic syndrome caused by TACE and is considered to be related to tumor necrosis, secondary inflammatory reaction, tumor size, and the amount of embolic material used.^[7,8]^ Although post-TACE fever is often mild and self-limited, it may not be significantly related to the long-term survival rate of patients after TACE, but it can significantly prolong the length of hospitalization and affect the follow-up treatment or reduce the patients’ confidence in repeated treatment.^[9,10]^ Nevertheless, few data have been published concerning PEF, especially in patients who underwent D-TACE; therefore, we investigated the risk factors and clinical significance of PEF that developed after TACE in patients with HCC, it is of great significance to accurately and timely judge the cause of fever after embolization, effectively communicate and dredge with the patients with fever after embolization, and improve the curative effect and the compliance of patients.

One of the multiple risk factors for PEF, as shown in the present study, is the size of the beads. It has been reported that huge particle size of embolized microspheres reduce post embolization syndrome including PEF,^[11]^ and the present study showed a similar finding. It has been reported that the lethality of microspheres is negatively related to its particle size, but positively related to its safety. The possible explanation for this phenomenon may be due to embolization-related tumor necrosis. The smaller the size of the microsphere is, the easier it is to enter the peripheral blood vessels in the liver. Peripheral embolic agents can pass through the physiological communicating branches and pathological arteriovenous short circuit between hepatic artery and portal vein or between hepatic artery and hepatic vein, and enter the pulmonary circulation with systemic circulation, causing pulmonary microinfarction and fibrosis. However, the multivariate analysis showed that the pre-TACE blood urea was independent risk factors, which is similar to the result of another recent study.^[12]^ There is no direct relationship between blood urea level and fever, but this study found that there is a negative correlation between them. The level of blood urea in the normal range is related to many factors, such as catabolism, protein intake, and so on. In this study, the consumption of tumor can lead to the increase of protein decomposition in the body, which can increase the level of blood urea, and the state of high protein decomposition can affect the synthesis of inflammatory substances and immune proteins, and then affect the reaction of inflammation, whether blood urea can influence post-TACE fever is rather controversial. Thus, further prospective studies should be conducted to confirm this finding.

PEF is a common clinical manifestation in postembolism syndrome caused by TACE, which can further reflect the degree of tumor necrosis and the effectiveness of embolization.^[13–16]^ In this study, by analyzing the recent postoperative outcome of all patients, we found that the short-term outcome of the hyperthermia group was better than that of the hypothermia group, which may be related to the complete vascular embolization of the tumor and its stabilization or even necrosis. The results of the study showed that patients with an abundant blood supply to the tumor, the absence of moderate to severe hepatic arteriovenous fistulas, adequate embolization of the tumor supply artery with drug-laden microspheres, and postoperative fever after liquefied necrosis in the center of the tumor were more likely to develop postoperative symptoms. Not surprisingly, after sufficient tumor necrosis, patients are prone to fever, but the enhanced portion of the tumor is significantly reduced, and according to the mRECIST criteria, this group of patients is treated with relatively good short-term efficacy. However, in this study, we did not observe a correlation between PEF and survival. Raoul et al^[17]^ suggested that these factors such as Child-Pugh score, reduced liver function, alpha-fetoprotein level, tumor size, tumor number, tumor type, portal vein thrombosis, multiple TACE sessions, and lobar embolization associated with poor TACE outcomes. In this study, prognostic factors including portal vein thrombosis, Child-Pugh stage, and cycles of DEB-TACE had influenced the clinical death. However, the difference with other studies is that iodized oil was not an independent factor affecting the fever of the patients. In our study, most of the patients enrolled used less iodinated oil during the use of drug-laden microspheres, further reducing the damage to liver function. Thus, there were relatively few patients with severe liver function impairment and liver function did not affect prognosis in this study.^[12]^ Fever was the manifestation of necrotic absorption after tumor treatment, which prolonged the hospitalization time of the patients, but had no effect on the survival time.^[18]^ The mean OS was 15.0 months (95% CI, 14.9–18.5) in the no fever group and 12.1 months (95% CI, 9.2–14.9) in the fever group (*P* = .192). These research results could not only help nurses infer the trend of postoperative fever based on intra-operative drug administration and laboratory examination data, but also could carry out predictive nursing, strengthen patient education and psychological nursing, and improve the effect of treatment.

The present study still has several limitations. First, this is a retrospective study with a relatively small number of patients. The results are warranted to confirm in large population-based prospective studies in the future. In addition, previous studies have found that the dose of lipiodol determines the extent of tumor tissue necrosis and is an important factor in fever; the duration of fever after TACE is positively correlated with the amount of lipiodol. As the focus of this study was to predict factors associated with fever, it did not explore whether lipiodol dosage was an independent pathogenic factor or a concomitant factor contributing to fever.

## 5 Conclusions

In conclusion, the pre-TACE blood urea and the size of the beads were independent indicators for fever after D-TACE in HCC patients. However, there were no relationships between PEF after TACE and survival.

## Author contributions

**Conceptualization:** Jinlong Song.

**Data curation:** Jinpeng Li, Congcong Shi.

**Formal analysis:** Jinpeng Li.

**Investigation:** Jinlong Song.

**Project administration:** Jutian Shi, Jinlong Song.

**Resources:** Jutian Shi.

**Software:** Congcong Shi, Jutian Shi, Nan Wang.

**Supervision:** Nan Wang.

**Validation:** Jinlong Song.

**Writing – original draft:** Jinpeng Li, Nan Wang.

**Writing – review & editing:** Jinpeng Li.
